# Lateralization in Alpha-Band Oscillations Predicts the Locus and Spatial Distribution of Attention

**DOI:** 10.1371/journal.pone.0154796

**Published:** 2016-05-04

**Authors:** Akiko Ikkai, Sangita Dandekar, Clayton E. Curtis

**Affiliations:** 1 Department of Psychology, New York University, New York, New York, United States of America; 2 Center for Neural Sciences, New York University, New York, New York, United States of America; Harvard Medical School/Massachusetts General Hospital, UNITED STATES

## Abstract

Attending to a task-relevant location changes how neural activity oscillates in the alpha band (8–13Hz) in posterior visual cortical areas. However, a clear understanding of the relationships between top-down attention, changes in alpha oscillations in visual cortex, and attention performance are still poorly understood. Here, we tested the degree to which the posterior alpha power tracked the locus of attention, the distribution of attention, and how well the topography of alpha could predict the locus of attention. We recorded magnetoencephalographic (MEG) data while subjects performed an attention demanding visual discrimination task that dissociated the direction of attention from the direction of a saccade to indicate choice. On some trials, an endogenous cue predicted the target’s location, while on others it contained no spatial information. When the target’s location was cued, alpha power decreased in sensors over occipital cortex contralateral to the attended visual field. When the cue did not predict the target’s location, alpha power again decreased in sensors over occipital cortex, but bilaterally, and increased in sensors over frontal cortex. Thus, the distribution and the topography of alpha reliably indicated the locus of covert attention. Together, these results suggest that alpha synchronization reflects changes in the excitability of populations of neurons whose receptive fields match the locus of attention. This is consistent with the hypothesis that alpha oscillations reflect the neural mechanisms by which top-down control of attention biases information processing and modulate the activity of neurons in visual cortex.

## Introduction

Spatial attention selects subsets of locations in cluttered environments that contain information relevant to the current or near-future goals. Attending to a particular location causes changes in neural activity across a number of brain regions. Previously, our functional magnetic resonance imaging (fMRI) studies showed that neural activity in portions of prefrontal cortex (PFC) and the posterior parietal cortex (PPC) persists during the maintenance of spatial attention and that activity is greater in the hemisphere contralateral to the attended hemifield [1]. In the context of similar data from both monkey electrophysiology and human neuroimaging studies, these results support the theory that neural population activity in PFC and PPC forms spatial maps of attentional priority [2–7].

The read-out of these priority maps may form the basis for biasing activity in visual cortex in favor of neurons whose receptive fields match the prioritized locations. Indeed, neural activity is enhanced in visual neurons when the locus of attention matches a neuron’s receptive field [8–12]. The influential biased competition model states that since neural representation in visual cortex is competitive, attention is needed to resolve the competition in favor of attended information [13]. When multiple stimuli are presented within a neuron’s receptive field, they appear to compete in a mutually suppressive manner [14–16]. In visual cortical areas, neural activity reflects this suppression and is diminished. Importantly, simply attending to one of the items releases the suppression and neural activity returns to levels similar to when the stimulus was presented alone [14, 15, 17, 18]. The read-out of priority maps by visual cortex may be the mechanism by which attention biases activity in favor of the neurons whose receptive fields contain the attended stimulus.

Recently, studies of humans using electroencephalograpy (EEG) and MEG have investigated how synchronous activity of populations of occipital and parietal neurons change with attention. In general, alpha (8–13Hz) power decreases over posterior sensors during the deployment of attention [19–25]. Specifically, attention to a particular location results in a decrease in alpha power in the hemisphere contralateral to the attended visual field [22–25], and an increase in power contralateral to the ignored visual field [26–28]. These results suggest that alpha oscillations might reflect the neural mechanisms by which top-down attention signals sculpt the activity gain of neurons in visual cortex. That is, a power decrease may indicate that different groups of neurons oscillate asynchronously from each other as a result of specialized stimulus processing. Consistent with this idea, alpha power decrease is correlated with enhanced target detection [20, 22, 25, 26, 29]. In addition to the performance enhancement, posterior alpha power decrease correlates with the amplitudes of N1, P1 or P2, early sensory event-related components [20, 30]. Such spatial selectivity of alpha oscillations was also observed in the somatosensory domain [31] where subjects expected a delivery of tactile stimulation via a visual cue.

In the present study, we tested the degree to which the posterior alpha power tracked the locus of attention and the distribution of attention. We recorded MEG while subjects performed an attention-demanding target detection task. We used two types of endogenous cues to manipulate the focus of attention: a predictive cue that indicated the target with 100% spatial certainty and a neutral cue that provided no information about the location of the target. Trial types were intermixed. Based on this manipulation, we tested specific predictions. First, a cue with predictive validity would result in focused attention directed at a single specific location in the visual field. The certainty of the target location will facilitate spatially selective visual processing and reduce competitive neural interactions. Therefore, we predicted decreased alpha power in the sensors in the hemisphere contralateral to the cued visual field. Second, a neutral cue provided only temporal information without spatial information about the target. Thus, spatial attention would be distributed among potential and competing target locations. We expected the effect of a spatially selective top-down attention to be smaller in uninformative compared to informative cue trials, which would result in smaller decrease in posterior pre-target alpha. Lastly, we tested whether alpha power increases in the hemisphere contralateral to the unattended visual field, as it is thought that such changes might be related to active suppression of potentially distracting stimuli. These predictions were designed to test the link between top-down attention and its effects on posterior sensory regions that underlies enhanced target processing at an attended location.

## Materials and Methods

### Subjects

We recruited 16 subjects (5 female, all right-handed, 22–42 years old) that each received compensation for participation. All reported normal or corrected-to-normal vision, and no history of neurological disorders. Subjects came in for a 1-hour practice session prior to the MEG experiment. Subjects gave written informed consent, and the human subjects Institutional Review Board at New York University approved all procedures.

### Behavioral Procedure

**Fig 1:**
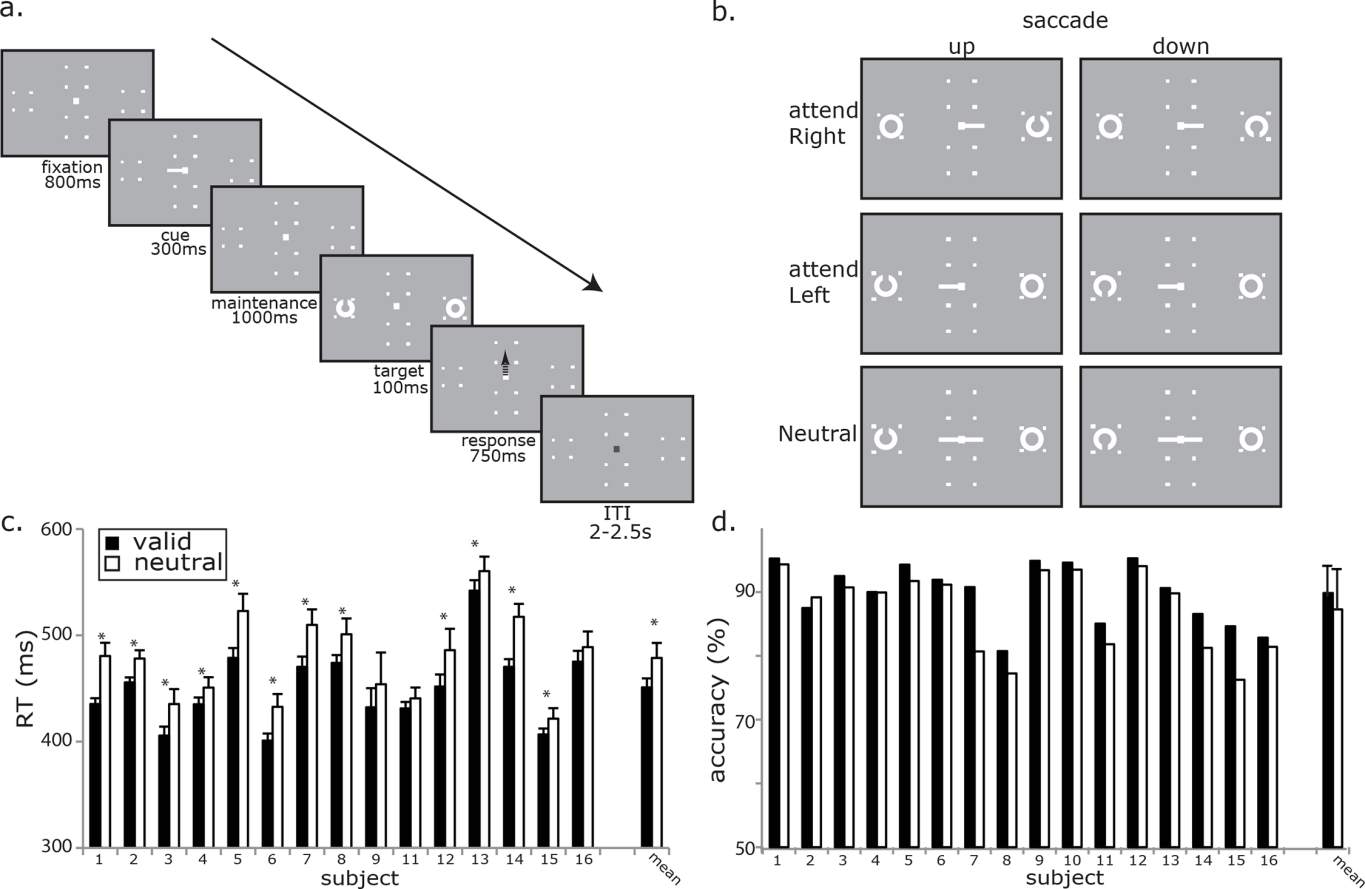
Trial schematic and behavioral results. a). Events during a valid trial. Subjects were cued to one visual field with an endogenous cue (100% validity). Subjects detected whether the target (Landolt ring) had a gap on the top or bottom, and made an eye movement to the corresponding placeholder. b). Trial and response types. Center cue informed which visual field would contain the target. A cue in Neutral trial pointed to the both visual fields, containing no directional information. c). Accuracy for each subject and mean. d). Saccade RT for each subject and mean. Error bars represent standard error.

All stimuli were controlled with MGL (http://justingardner.net/mgl), and projected (InFocus LP425) into the magnetically shielded room (Vacuumschmelze, Hanau, Germany) onto a screen placed 38 cm away from subjects. Trial schematics are illustrated in Fig 1A. Subjects fixated a small white dot in the center of the screen for 800ms at the beginning of the trial. A cue (300 ms) and a 1000 ms delay followed, after which two white circles, one on each visual field (6 degrees of visual angle from fixation point), appeared on the horizontal meridian for 100 ms. Subjects were instructed to determine whether the small gap in one of the circles was on the top or bottom (i.e. the target was a Landolt ring). If the gap was at the top portion of a Landolt ring, the correct response was a saccade to the target located at the top visual field along the vertical meridian (6 degrees) within 750 ms. On the other hand, if the gap was at the bottom of the Landolt ring, subjects were instructed to make a saccade to the bottom visual field. This procedure allowed us to disassociate motor planning processes associated with the direction of the saccade from the location of the targets attended. Two to 2.5 sec of intertrial interval (ITI) followed and a new trial began. There were two cue types: one that predicted the target’s location (“valid” cues; 71.5% of trials) and one that was uninformative about the target’s location (“neutral” cues; 28.5%). The valid cue was a white bar (0.5 degrees of visual angle) pointing either left or right from the fixation dot, with 100% validity. Subjects were told explicitly of the validity of the cue before the data collection, and therefore were encouraged to pay attention to the cued visual field. The neutral cue was the same white bar, pointing to both visual fields (1 degree, centered at fixation, Fig 1B). Twenty percent of the valid cue trial was with short delays (random between 250 ~ 550 ms). These trials were included in order to encourage subjects to shift attention as fast as possible, and to add jitter to the delay length. Because of the small number of trials and the contamination due to the onset/offset of the cue, these trials were excluded from analysis. Cue and trial types, as well as the location of the gap (top/bottom) were randomized. Subject performance was monitored throughout the experiment, and the gap size was adjusted to maintain an overall high performance (85% accuracy). Subjects performed 10 blocks, each of which consisted of 56 trials and lasted about 5 min.

### MEG recording and preprocessing

A 157-channel axial gradiometer MEG system (Kanazawa Institute of Technology, Kanazawa, Japan) was used to collect data with a sampling frequency of 500 Hz. Head position locator coils were used to localize the head location in the MEG. MEG data were first noise-reduced in the MEG160 (Yokogawa Electric Corporation and Eagle Technology Corporation, Tokyo, Japan), using the continuously adjusted least-squares method [32]. We analyzed MEG sensor data using Fieldtrip (http://fieldtrip.fcdonders.nl/). Data was first high-pass filtered at 1Hz, and then segmented into epochs between 2000ms before and 2000ms after the onset of the delay period. Independent component analysis (ICA) was performed on the epoched data, and eye blink component was identified and removed from the data. Epochs were excluded from further analysis if the spike artifacts were detected visually, or the variance of sensor activity was above 2SD away from the mean within an epoch.

### MEG Time-frequency analysis

**Fig 2:**
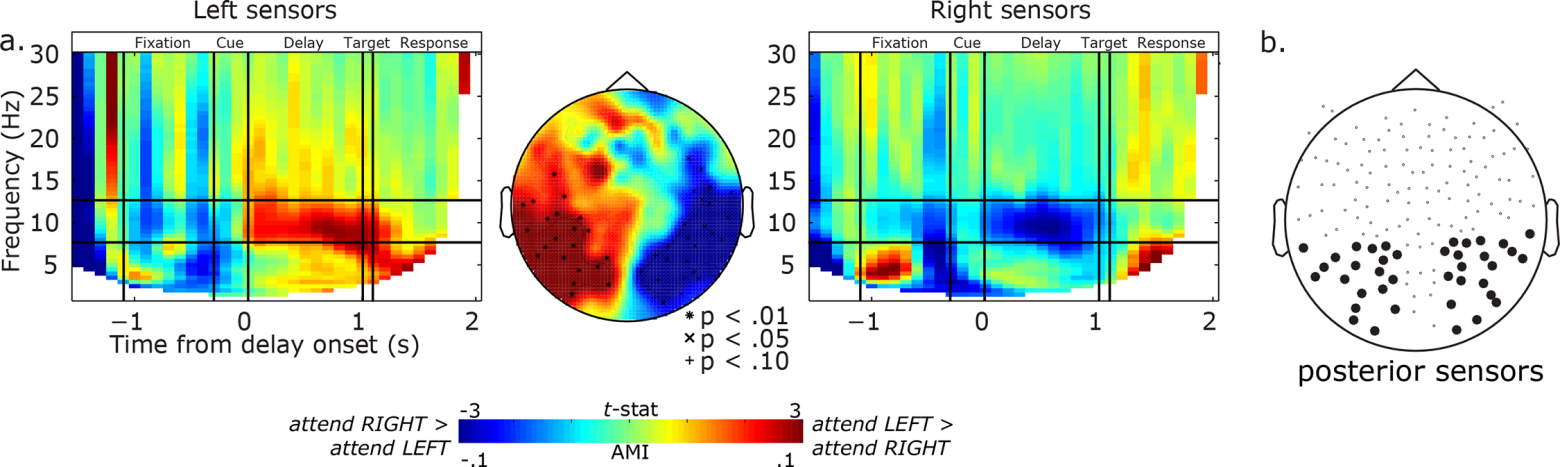
Attend Left vs. attend Right contrast. a). Topography of t-statistics obtained from AMI against 0 (middle). Left side of the topography corresponds to the left hemisphere throughout this article. Significantly modulated sensors are marked with symbols. TFR of AMI averaged across the posterior left sensors (left panel) and right sensors (Right panel). Warm color indicates higher alpha power in attend Left than attend Right conditions, and cool color indicates opposite pattern. b). Selected posterior sensors (20 sensors/hemisphere). These sensors were selected based solely on their locations.

MEG data were converted into planar gradients. Axial gradients represent each source with a bipolar magnetic field flux pattern associated with its neural current. A cluster-based nonparametric permutation test (see below) performed on this bipolar distribution would result in two clusters associated with one neural source. Planar gradients, on the other hand, express the power of a source that lies beneath as a single peak [33]. A permutation test would localize a single cluster around the peak of activation, thus making the interpretation of data more straightforward and meaningful. In order to perform this conversion, we first obtained the estimates of the horizontal and vertical planar gradients at each sensor, based on the signals from neighboring sensors [33] before time-frequency analysis. For our the time-frequency analysis in Fig 2, we used a multitaper approach with a sliding time window with the width of 5 cycles/frequency, at a step size of 50ms, and a spectral smoothing of ±.5 Hz around each frequency of interest (1, 1.5, …29.5, 30Hz) to estimate the time-course in power separately for horizontal and vertical component of signal for each sensor. Horizontal and vertical planar gradients were combined after the spectral analysis. For each subject, we averaged spectral power for each attention condition separately. In order to estimate the spatial selectivity of power changes, we then calculated an Attention Modulation Index (AMI) at each sensor as follows: AMI = (attend Left vs. attend Right)/(attend Left + attend Right). AMI is an index of spatial selectivity at each sensor. A positive AMI would result from higher power in the attend Left than the attend Right condition. On the other hand, a negative AMI would result from higher power in the attend Right than the attend Left condition. Additionally, in order to estimate the amplitude of power changes we normalized power with respect to the baseline fixation period at each sensor as follows: (attention delay–baseline)/(attention delay + baseline). This is a similar procedure to obtaining BOLD percent signal change from baseline in our previous MRI studies [4, 34, 35]. We performed this calculation for each trial type (attend Left/Right and Neutral).

Based on our *a priori* hypothesis, which focused on posterior alpha, we selected a subset of posterior sensors from the left and right hemisphere separately for further analysis (Fig 2B). This procedure was purely based on the sensor location, and thus, the most unbiased and conservative method to select sensors. We selected all posterior sensors to the left and right of midline. AMI was averaged across subjects, and averaged across these selected posterior sensors, to produce time-frequency representations (TFRs, Fig 2A).

### Statistical test in topography

**Fig 3:**
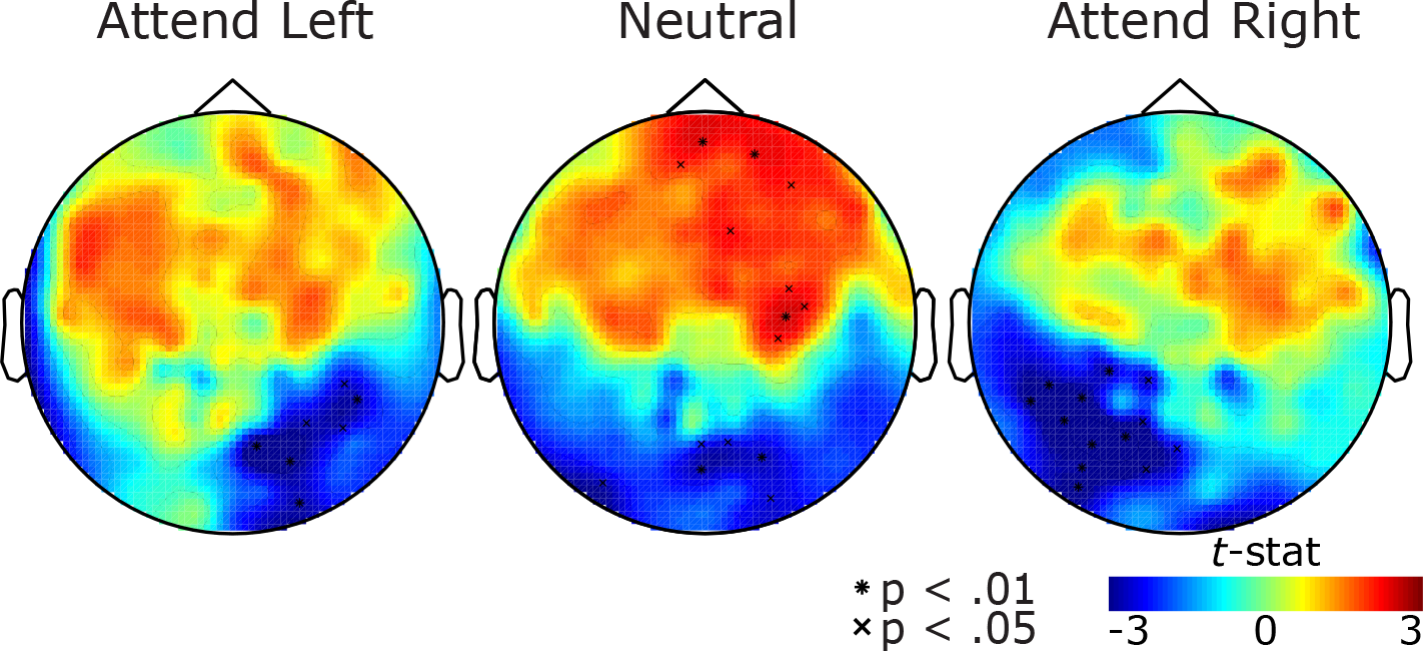
Pre-target alpha during the delay period (500 to 1000 ms) relative to baseline period (-800 to -300 ms). Topographies of t-statistics obtained from delay vs. baseline contrast. Warmer and cooler color indicates sensors with increased and decreased alpha power during the delay compared to baseline, respectively, with significant sensors marked with symbols

We used a nonparametric randomization test [36] to statistically test the difference between conditions (Fig 2A, middle) and between attention delay and the baseline (Fig 3A). This procedure controls for type I error by calculating the cluster-level statistics by randomizing trial labels on each iteration. First, in order to compare between attention conditions (attend Left vs. attend Right), AMI were averaged over the time (500–1000 ms of delay) at an alpha frequency that we definded as 10Hz, and t-statistics were obtained at each sensor comparing the AMI against 0. On each iteration, clusters of sensors below an alpha of 0.05 were identified, and their t-values were summed. The largest sum of t-values was used as a t-statistic. This procedure was repeated 500 times to create the null distribution. The p value was estimated according to the proportion of the null distribution exceeding the observed cluster-level test statistic. In the case of a contrast between attention delay (for example, attend Left) and the baseline, we calculated a similar index as AMI and used it in the nonparametric randomization test. That is, for each subject, we first averaged power spectrum for attend Left condition. Then we calculated the average power across time (-800 to -300 sec for baseline, and 500 to 1000 ms sec for attend Left delay; see Fig 1A for trial timing) at an alpha frequency that we defined as 10Hz. These values were used to calculate the index such as (attend Left—baseline)/(attend Left + baseline). This index was compared against 0 to obtain t-statistics at each sensor during the nonparametric randomization test as described. Same procedure was repeated for the attend Right and Neutral conditions.

### Eye movement recording and analysis

Eye movements were recorded at 1kHz throughout the experiment using an MEG compatible fiber optic EyeLink 2K (Ontario, Canada). Subjects were told to refrain from blinking and unnecessary eye movements during the trial until ITI. The vertical component of eye movement was transformed to degrees of visual angle, calibrated using a third-order polynomial algorithm that fit eye positions to the fixation and saccade target positions, and scored offline with in-house software. Any trials with unwanted/incorrect saccades (e.g. primary saccade to wrong directions, breaking fixation during the delay), and trials with no response were discarded from further analysis. Only trials in which the primary saccade landed on the correct target were further analyzed. Saccadic response times were estimated with semiautomatic routines that relied on the velocity of the eye reaching 30°/s to determine the onset of saccades. The data were also inspected visually, trial by trial, and any over/underestimation of saccade initiation point was corrected manually. During the post-experimental interview, it was revealed that one subject misunderstood the timing of the response and waited until the response period was over to respond. Since this subject’s accuracy was not different from others, we included this subject in the accuracy analysis and topography display. Due to technical problem, we were not able to determine saccade onset time (reaction time: RT) for one of the subject. For this subject, saccade endpoints were used for accuracy calculation.

## Results

### Behavioral performance

Accuracy and response times are shown in Fig 1B and 1C, respectively. Subjects performed with high accuracy that was near the predetermined threshold (M = 87.53%, SD = 5.32). Consistent with past studies of endogenous guidance of attention, compared to the neutral condition with high uncertainty (M = 85.78%, SD = 5.86), subjects performed significantly better when the target was cued and they attended Left (M = 88.48%, SD = 4.95, paired-*t*(15) = 3.37, p < .005) or attended Right (M = 88.32%, SD = 5.02, paired-*t*(15) = 2.44, p < .03). There was no significant difference between attend Left and attend Right conditions (paired-*t*(15) = .15, p > .05). After removing 2 subjects’ data (see Methods), an analysis of RTs also showed that performance was enhanced by the spatial certainty of the valid cue. Compared to uninformative neutral cue (M = 480.51ms, SD = 42.53), RT was significantly faster in attend Left (M = 449.84ms, SD = 41.62, paired-*t*(13) = -8.89, p < .001) and in attend Right (M = 453.41ms, SD = 39.11, paired-*t*(14) = -7.09, p < .001). As with accuracy, there was no significant difference in RT between attend Left and attend Right (paired-*t*(14) = -1.16, p > .05). Our behavioral results confirm our manipulation of attention with our cueing paradigm and provide basis for the interpretation of our MEG data in terms of the top-down influence on task-relevant visual processing.

### Pre-target alpha power

Our primary aim was to examine how spatial attention affects alpha oscillations in visual cortex. Thus, we first ensured that our task-manipulation had significant effects on alpha band power during the attention maintenance period. In the middle panel of Fig 2A, we plot the effects that attention has on the topographic pattern of alpha band activity during the maintenance delay by computing t-statistics comparing AMI to zero. In the side panels of Fig 2A, we plot TFRs of the AMI averaged across the selected posterior sensors over the left and right hemispheres separately (Fig 2B). From these data, it is clear that alpha power changes according to the direction of attention, especially in the posterior sensors over visual cortex. In the left sensors, alpha power decreases when attention is directed into the right visual field compared to the left. Similarly, in the right sensors, alpha power decreases when attention is directed into the left visual field compared to the right. Relative changes in power are largely confined to the alpha band and are limited to and persist throughout the temporal epoch in which attention is being maintained in the periphery.

The spatiotopographic map of alpha power changes suggests that alpha power both decreases in the hemisphere contralateral to the attended visual field and increases in the hemisphere ipsilateral to the attended visual field (Fig 2A). Of course, such a lateralization could results from contralateral power decrease, ipsilateral power increase, or from a combination of both. In order to clarify the direction of the effect, we contrasted pre-target alpha power with that from baseline fixation period. When attention was deployed to the left or right, alpha power decreased in posterior sensors contralateral to the attended hemifield (Fig 3; p < .05, corrected). However, we did not find that alpha power increased in posterior sensors ipsilateral to the direction of attention. Therefore, attention causes mainly a desynchronization of contralateralized alpha oscillations over visual cortex. In the Neutral condition, where we found a decreased alpha power in the posterior sensors bilaterally and an increased alpha power in the anterior sensors bilaterally, (Fig 3; p < .05, corrected). The increase in anterior alpha may reflect the increased attention demands in the Neutral relative to the valid cue conditions.

**Fig 4:**
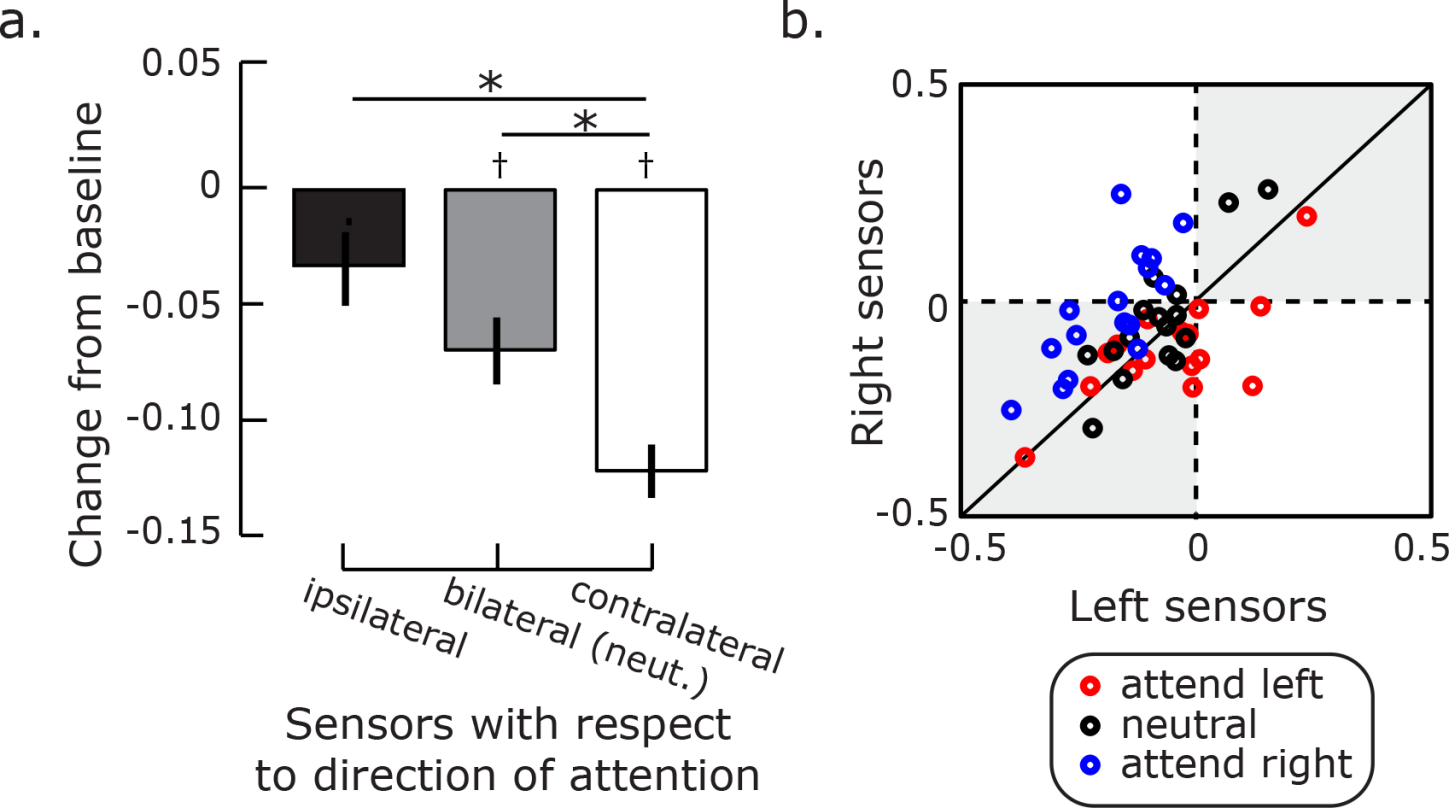
Effects of focused and distributed attention on posterior alpha. a). Mean power change during the attention delay from baseline was computed for posterior sensors ipsilateral or contralateral to the cued visual field (left and right bars), and separately for bilateral sensors following the uninformative cue (middle bar) when attention is presumably distributed across both visual fields. Error bars represent sem. The * symbol indicates a significant difference in alpha power change between the conditions, while the † symbol indicates that the alpha power change was different from the pretrial baseline. b). Scatter plot showing alpha power change from baseline for each trial type, where x and y-axes represent averaged power change extracted from the left and right posterior sensor groups. Each dot represents one subject. Note how the blue and red dots mostly lie above and below, respectively, the unity line highlighting the lateralization of alpha power changes.

Our hypothesis was that top-down spatial attention control affects neural synchrony in the posterior visual areas where task-relevant information was processed. Therefore, we tested the degree to which alpha power is modulated in a retinotopically specific manner. For each trial type (attend Left/Right and Neutral), we estimated the change in alpha power during the attention delay relative to the baseline period. A repeated measure ANOVA showed the predicted significant interaction between alpha power in the hemisphere (Left/Right sensor groups) and the attended visual field (Left/Right/Neutral), F(2,14) = 11.29, p < .001. Not surprisingly, the main effects of hemisphere, F(1,15) = 3.08, p = .07, and attention, F(2,14) = 0.86, p = .43, were not significant. To unpack the significant interaction, we combined the data from the two hemispheres as a function of the direction of attention. For each subject, we estimated the mean alpha power change from the pretrial baseline in the sensors contralateral to the attended visual field, ipsilateral to the attended visual field, and bilateral in the neutral condition when attention was directed to both visual fields. An ANOVA indicated that there was a significant linear effect of attention on the distribution of alpha power, F(1,15) = 18.26, p < .001 (a quadratic test was not significant, p = .44.) Follow-up contrasts revealed that alpha decreased significantly compared to baseline in sensors bilaterally when attention is distributed in the neutral condition (t(15) = -2.49, p < .03) and in the sensors contralateral to the attended visual field (t(15) = -5.31, p < .001). Although alpha did not significantly differ from baseline in sensors ipsilateral to the attended visual field (t(15) = -1.37, p -.19), it clearly did not increase (Fig 4A). Notably, the informativeness of the cue had a significant effect on the magnitude of alpha power decrease. When the cue indicated the visual field of the upcoming target, alpha power decreased more in the contralateral hemisphere than in the neutral condition where attention was presumably spread across the two visual fields (p < .04, corrected; Fig 4A).

Across subjects, there was substantial variability in the changes in alpha power (Fig 4B). In all conditions, there was a significant correlation between the modulation of alpha power in the left and right sensors (all r’s > .70, all p’s < .003; df = 14). With attention, the majority of subjects showed a decrease in power in both sensor groups (bottom-left quadrant), and only a few showed an increase in power in both sensor groups (top-right quadrant). Furthermore, in the attend Right condition, none of the subjects showed an increase in power in both sensor groups, but all of the subjects showed a decrease in power in the posterior left sensors. In addition, 60% of the subjects showed an increase in power in the posterior right sensors (top-left quadrant). Overall, alpha power decreased in a graded fashion with the sensors contralateral to the attended visual field resulting in the largest power decrease. This decreased may be reflecting the amount of attentional resources allocated in each visual field. Moreover, we did not find that alpha power increases in the hemisphere ipsilateral to the direction of attention.

## Discussion

The present study aimed to measure the effects of spatially specific top-down attention on neural activity in visual cortical areas. We used an endogenous cueing task in which subjects covertly attended the location of the upcoming target to facilitate discrimination of the orientation of a gap in a Landolt ring. Importantly, subjects indicated the orientation of the gap by making a saccade upwards or downwards, directions that were orthogonal to the direction of attention. This allowed us to dissociate the direction of covert attention from the direction of the motor command to indicate choice. We observed relative decreases in alpha power in sensors over posterior cortex contralateral to the attended visual field. To better understand the effect of top-down attention control, we included a Neutral condition, where the endogenous cue was not informative about the target’s location. When attention was distributed across both visual fields, we observed an alpha power decrease bilaterally in posterior sensors. We will discuss the possible role of alpha oscillations in attention in the context of the following main findings of the current study. First, alpha oscillations desynchronized in the hemisphere of the occipital lobe that was contralateral to the attended visual field. Second, varying the spatial certainty with which attention could be directed affected the topography of alpha power in sensors over occipital cortex. Lastly, we find that alpha power did not increase in the hemifield contralateral to the unattended visual field; if anything it decreased.

### Alpha desynchronization contralateral to the attended hemifield

When attention was deployed to one of the lateralized targets, alpha power systematically changed in MEG sensors over occipital cortex. Notably, these changes occurred prior to the onset of potential target stimuli. They began shortly after the onset of the endogenous cue that was used to select the location to attend. Moreover, they persisted as long as attention was covertly maintained. These changes in synchronous neural activity were not visually evoked, but rather reflect the deployment and maintenance of attention. Moreover, the changes in alpha power were lateralized in the brain with respect to the direction of attention. Alpha oscillations desynchronized in the hemisphere of the occipital lobe that was contralateral to the attended visual field. Although spatial source localization is imprecise with MEG, our changes in alpha power over the contralateral visual cortex are consistent with numerous fMRI studies that have demonstrated that attention causes widespread lateralized changes in occipital and posterior temporal areas [6, 18, 37–40], as well as critical roles in object perception [34, 41–46]. Moreover, our results are inline with human fMRI studies that show increased activation in extrastriate visual areas when subjects expect the delivery of the visual stimuli, even with absence of visual stimuli [47–51].

Like in the present study, other studies have found that alpha power decreases in the hemisphere contralateral to the attended visual field, the location of working memory representation, and the location toward which a motor response is planned [23, 31, 52, 53]. Decreased power at the sensor level reflects large-scale desynchronization of oscillatory rhythms among the underlying populations of neurons. Increased activity in local neuronal networks associated with the encoding of visual information into a neural representation may desynchronize the population activity [54]. In the present study, we found alpha desychronization during the maintenance of attention prior to the target’s appearance. Therefore, we propose that spatially specific top-down signals, in the absence of retinal input, were sufficient to disrupt synchronous alpha band activity in visual cortex. Presumably, attention increased the excitability of neurons with receptive fields that matched the locus of attention [8, 10–12, 55–57].

In the current study, the changes in the lateralization of alpha power with respect to the direction of attention were driven mainly by decreases in the hemisphere contralateral to the attended hemifield, and not by increases in the hemisphere contralateral to the nonattended hemifield noted in other studies [26, 28, 29, 53, 58, 59]. In these studies, increased alpha power reflects inhibition of non-attended or irrelevant information. The authors of these studies argue that synchronous neural activity in the alpha range relates to a gating mechanism that limits computational resources from being directed towards task-irrelevant regions (i.e. unattended hemifield) [26, 60, 61]. We suspect that task differences may underlie the lack of increased alpha power in the ipsilateral hemisphere. Alpha power increase is often observed in tasks where there are competing stimuli that need to be actively suppressed, such as visual stimuli in the other visual field [26, 28, 62], competing modality[61], competing sites of stimulus delivery [59, 63], or even differently during the shift and maintenance of spatial attention [64]. Under the current task design where the endogenous cue was 100% valid and there were only stationary placeholders during the delay period, the best strategy was to allocate resource to the cued visual field undividedly. Critically, the need to inhibit processing in the non-attended hemifield was very low given that nothing but the placeholders were visible during the attention delay. Additionally, covert attention was needed in order to detect the orientation of the gap in the Landolt ring. Even if the stimulus was visible or subjects anticipated its appearance [48], the non-attended ring would not compete with the discrimination since it has no gap that might lead to an alternative response. We believe this factor not only explains why we did not observe alpha power increases ipsilaterally, but also helps clarify the boundary conditions within which alpha increases related to inhibition may exist.

Interestingly, we noted a few asymmetries in how spatially-directed attention modulated alpha in the two posterior cortical hemispheres. The direction of attention had an overall larger effect on the changes in alpha power in the sensors over the left compared to right hemisphere. Decreases in alpha power were larger in the left compared to right hemisphere when attention was directed into the contralateral visual field. In the right compared to the left hemisphere, alpha desynchronization was not as lateralized as a function of the attended visual field. Similarly, attending to the right evoked a strongly lateralized pattern of decreases in alpha in the left hemisphere and increases in alpha in the right hemisphere. Attending to the left evoked a weakly lateralized change in alpha power. Finally, on an individual basis more subjects showed a relative increase in alpha power in the right than left cortex. Haegens et al (2011) reported a similar asymmetry. They reported a disproportionally larger alpha decrease in sensors over the left compared to right sensorimotor cortex when subjects expected the delivery of a tactile stimulus to the contralateral hand. However, in Haegens et al, subjects made button press responses with their right hand, and therefore motor preparatory processes may have influenced those results. In the present study, subjects made eye-movements up or down, saccades that were orthogonal to the left/right direction of the attended targets. Thus, the hemispheric asymmetries reported in Haegens et al and the present study are unlikely to stem from motor preparatory processes and are not specific to any one motor modality. It is tempting to conclude that the asymmetries that we noted are related to the right hemisphere dominance model of attention. This model attempts to explain why hemineglect is much more frequent after damage to the right compared to the left parietal cortex [65–67]. Indeed, the less lateralized changes in alpha power in the right hemisphere is consistent, at least, with the model’s assertion that the right hemisphere may play important roles in attention across both hemifields. An alternative to this theory, the Interhemispheric competition theory [68], posits that a mutual inhibition between hemispheres is fundamental to the control of spatial attention and the disruption of this balance between the hemispheres, following damage to the dorsal attention network, results in hemineglect [65, 69–72]. Indeed, recent work has shown that fMRI activity is largely contralaterally biased by the direction of attention regardless of the hemisphere [73]. Moreover, the degree of neural bias predicts individual differences in the degree to which spatial attention is naturally biased behaviorally and this bias can be altered by perturbations to retinotopic areas in the dorsal attention network [74]. Yet, we are still faced with trying to answer the question of why hemineglect is much more frequent after damage to the right hemisphere.

### Topography of alpha power modulated by the distribution of attention

In trials in which the cue was informative, attention could be focused on the target’s forthcoming location with 100% certainty. In other trials the cue was not informative about the target’s location and attention must be spread or distributed among the potential targets in each hemifield. As described above, when attention was focused alpha power decreased in MEG sensors over the occipital hemisphere contralateral to the attended hemifield. In contrast, when attention was distributed broadly across the visual field, the distribution of decreased alpha power over the visual cortex was concomitantly broad across both hemispheres. Previous studies have also found changes in the distribution of posterior alpha decreases with attention variables [20, 31]. Similar to the present study, Gould et al (2011) cued subjects endogenously to one visual field each trial to perform a target discrimination task. They found a significant linear decrease in posterior alpha power contralateral to the attended visual field as a function of the predictive validity of the cue. Similarly, Haegens et al (2011) visually cued subjects to expect a delivery of a tactile stimulation with the varied cue validity. They reported a significant linear effect on alpha lateralization based on the cue validity, which was mainly driven by a decrease in power in sensors contralateral to the cued side. These studies and our results strongly suggest the role of anticipatory alpha band neural synchronization in shaping underlying neuronal groups’ excitability. Furthermore, the fact that Haegens et al (2011) found a lateralization in sensor groups over somatosensory cortex, though subjects were cued visually, indicates that the neural changes indexed by alpha power can be directed by top-down mechanisms to behaviorally relevant sensory processing areas.

### Frontal alpha increases during spatially ambiguous trials

In addition to the bilateral decrease in posterior alpha, when the location of target was uncertain we observed increases in alpha power in MEG sensors over frontal cortex. Indeed, other studies have reported increased alpha power during the deployment of attention that localized to the prefrontal cortex [75, 76]. Additionally, fMRI studies have repeatedly shown that the activity of prefrontal cortex increases during the deployment of spatial attention [1, 4, 47, 48, 77–80]

Does alpha power in posterior cortical areas reflect different neural mechanisms than those in the prefrontal cortex? As described above, in the posterior cortex alpha power decreases are thought to index the desynchronization of neural activity due to stimulus specific neural processing, while increases are thought to reflect spatially specific inhibitory processes. It is unlikely that the increases in alpha that others and we have observed in prefrontal cortex reflect inhibition of local neural activity. Indeed, human EEG and MEG studies typically show that alpha power increases in the prefrontal cortex when task demands are increased, such as when working memory load increases [81–84] or when stimulus-response mapping is made more difficult [85]. In the context of the current study, attentional demands are greater when the cue is not predictive compared to when it is predictive of the target location. They are greater because the subject must monitor two locations for the potential target, which raises two intriguing possibilities. First, attention might be distributed equally to the potential target locations at the same time. Behavioral [86, 87] and neuroimaging [88, 89] studies support the idea that attention can be split into multiple “spotlights” or foci. In this framework, our subjects may have needed to split their attention between the two potential target locations. Such a distribution of attention places greater demands on attentional resources much like maintaining more than one item in mind places greater demands on working memory resources. Alternatively, a single focus of attention shifts back and forth between potential target locations rapidly. A recent behavioral study showed that when the target locations spread across both visual fields, the visual system samples both locations at theta rhythm [90]. Either of these mechanisms places greater demands on the neural mechanisms that control the allocation of attention and could account for the increases in alpha power. Unfortunately, the current study was not designed to test between these alternatives and we do not have evidence to support either of these possibilities. However, a behavioral study designed to dissociate these two hypotheses found that the periodic sampling model accounted for attention performance more reliably, even when there was only one target present [91].

## Conclusion

Spatial attention selects locations that contain goal-related information from among competing information in our cluttered visual world. As a result, our visual abilities are enhanced and the synchronous activities of neurons in the visual cortex change as a function of the locus of attention. These neural changes reflect the effects that attention have on neurons in the visual cortex, which are presumably caused by top-down attention controlling signals emanating from the frontal and parietal cortices. Here, we show that these attention signals sculpt the spatial topography of synchronous activity in the alpha band (8–13Hz) across the posterior visual cortex. Desynchronization of alpha activity in the hemisphere contralateral to the attended hemifield was a reliable predictor of the direction of attention and the spread of attention. Our findings provide evidence that the deployment of spatial attention leads to a change in the oscillatory patterns in the neurons of the sensory cortex. The desynchronization of populations of neurons may be caused by target-specific activity associated with the processing of visual information within the locus of attention. Therefore, alpha synchronization may reflect inhibitory mechanisms that suppress processing in irrelevant or unattended portions of the visual field.
